# Assessing Molecular Docking Tools to Guide Targeted Drug Discovery of CD38 Inhibitors

**DOI:** 10.3390/ijms21155183

**Published:** 2020-07-22

**Authors:** Eric D. Boittier, Yat Yin Tang, McKenna E. Buckley, Zachariah P. Schuurs, Derek J. Richard, Neha S. Gandhi

**Affiliations:** 1 Cancer & Ageing Research Program, Institute of Health and Biomedical Innovation at the Translational Research Institute (TRI), Queensland University of Technology (QUT), Brisbane, QLD 4102, Australia; boittier@qut.edu.au (E.D.B.); yatyin.tang@connect.qut.edu.au (Y.Y.T.); zachariah.schuurs@hdr.qut.edu.au (Z.P.S.); derek.richard@qut.edu.au (D.J.R.); 2 School of Chemistry and Physics, Faculty of Science and Engineering, Queensland University of Technology, Brisbane, QLD 4000, Australia; mckenna.buckley@connect.qut.edu.au; 3 School of Mathematical Sciences, Faculty of Science and Engineering, Queensland University of Technology, Brisbane, QLD 4000, Australia

**Keywords:** molecular docking, scoring functions, drug design, enzyme inhibitor, CD38 (cluster of differentiation 38 protein)

## Abstract

A promising protein target for computational drug development, the human cluster of differentiation 38 (CD38), plays a crucial role in many physiological and pathological processes, primarily through the upstream regulation of factors that control cytoplasmic Ca^2+^ concentrations. Recently, a small-molecule inhibitor of CD38 was shown to slow down pathways relating to aging and DNA damage. We examined the performance of seven docking programs for their ability to model protein-ligand interactions with CD38. A test set of twelve CD38 crystal structures, containing crystallized biologically relevant substrates, were used to assess pose prediction. The rankings for each program based on the median RMSD between the native and predicted were Vina, AD4 > PLANTS, Gold, Glide, Molegro > rDock. Forty-two compounds with known affinities were docked to assess the accuracy of the programs at affinity/ranking predictions. The rankings based on scoring power were: Vina, PLANTS > Glide, Gold > Molegro >> AutoDock 4 >> rDock. Out of the top four performing programs, Glide had the only scoring function that did not appear to show bias towards overpredicting the affinity of the ligand-based on its size. Factors that affect the reliability of pose prediction and scoring are discussed. General limitations and known biases of scoring functions are examined, aided in part by using molecular fingerprints and Random Forest classifiers. This machine learning approach may be used to systematically diagnose molecular features that are correlated with poor scoring accuracy.

## 1. Introduction

Chronological aging is related to a range of metabolic and pathophysiological changes. One such change is the age-related decline of intracellular levels of nicotinamide adenine dinucleotide (NAD) which has been linked to diseases such as obesity, diabetes, heart failure, inflammation, and cancer [1]. NAD, found in all living cells, is involved in metabolic redox reactions, cell signaling, and regulating levels of intracellular Ca^2+^. One important endogenous regulator of intra- and extracellular NAD levels is the human cluster of differentiation 38 protein (CD38) [2]. CD38 is a transmembrane protein that works to catalyze the degradation of NAD into adenosine diphosphate ribose (ADPR) or cyclic ADPR (cADPR) (cellular messengers for Ca^2+^ signaling), and to hydrolyze cADPR to ADPR. In a mouse model, inhibiting CD38 using a small-molecule inhibitor was linked to the inhibition of pathways relating to aging and the reduction of telomere-associated DNA damage. Over 200 small molecule inhibitors of CD38 have been reported in the literature. These compounds vary in class (NAD analogs, flavonoids, heterocyclic, etc.) and mechanism of action (covalent vs. non-covalent inhibition). Despite many promising drug candidates, taking a drug to market for CD38 targeting therapies remains challenging, due in part to off-target interactions and generally poor specificity.

**Figure 1:**
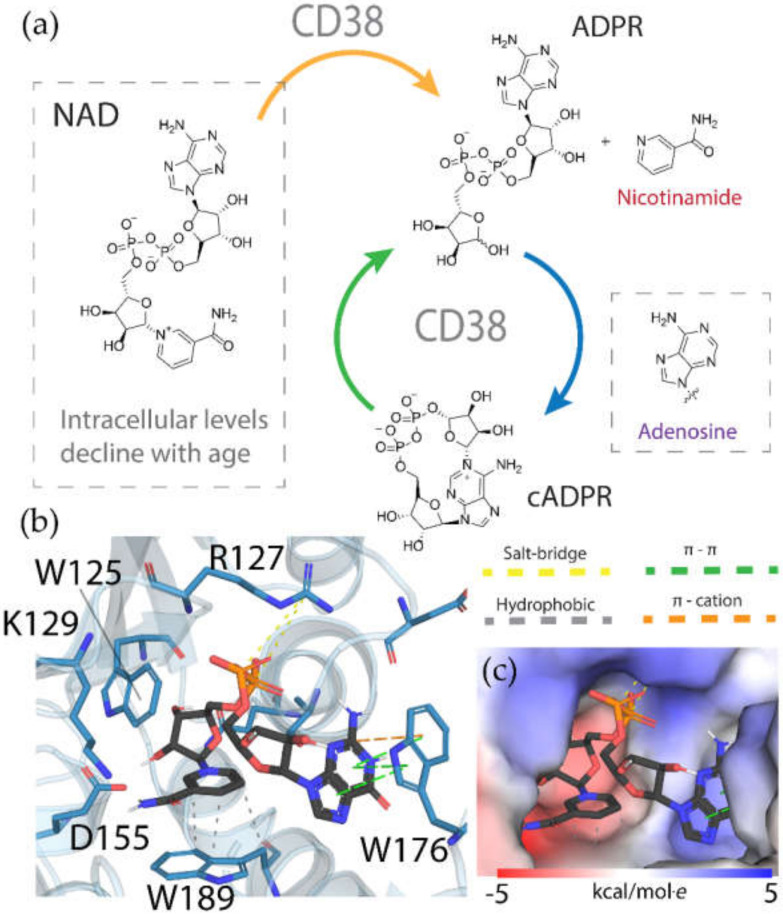
(**a**) Chemical transformations performed by CD38 involving important biomolecules such as NAD, ADPR, and cADPR. (**b**) Key interactions between CD38 and NAD (PDB code: 2O3U). Yellow, green, grey, and orange dotted lines represent salt-bridge, π–π, hydrophobic and π–cation interactions, respectively. A Van der Waals surface representation of the binding site (**c**), colored by the electrostatic potential, illustrates the shape and charge complementarity of the active site.

Crystal structures of CD38 co-crystallized with NAD, as well as cADPR and ATP-ribose, have been vital in determining the key catalytic residues [3]. These structures reveal the formation of hydrogen bonds between NAD and NAD-like ligands with E226 and R127, and Van der Waals interactions between W125, W176, and W189 (see Figure 1) [4]. The W189 indole ring forms π–π interactions with aromatic rings of the NAD substrate [5]. The mutation E226A eliminates the catalytic activity of CD38 [6]. The catalytic pocket demonstrates remarkable recognition of these substrates and undergoes substantial structural changes or “induced fit” upon binding. CD38 has a solvent accessible binding volume of about 986 Å^3^ when unbound, and 940 Å^3^ with the cADPR intermediate (PDB codes: 1YH3 and 2O3R, respectively; volumes were calculated using the CASTp (Computer Atlas of Surface Topography of Proteins) server) [7]. Previous computational efforts to model small molecules in complexes with CD38 have included molecular docking (referred to hereafter as ’docking’) [5,8,9,10,11,12]. Comparative Molecular Field Analysis (CoMFA) has also been previously used to inform CD38 drug development efforts with robust results [4]. However, these results cannot be generalized to other scaffolds, unlike docking [13].

Docking is a computational approach at predicting the binding pose and affinity of a small molecule ligand and protein. Docking involves two interrelated operations—a search algorithm and a scoring function. The performance of a docking program (or scoring functions in general) is usually assessed by three tests of increasing difficulty—docking, scoring, and screening. A ’docking’ test assesses the program’s ability to reproduce experimental crystallographic poses. Generally, this is done in a process called ’re-docking’, where the native ligand is docked to the binding site in a protein. Tests of the ’scoring poor’ assess the correlation between predicted affinities and experimentally determined affinities—either through regression (using Pearson’s correlation coefficient) or through ranking (where Spearman’s correlation coefficient is the statistic of interest) [14]. ’Screening’ power tests for the ability to determine true versus decoy binders, usually achieved by screening large libraries of compounds; however, this test is outside the scope of this paper. Previous investigations have suggested that the performance of a docking program is dependent on the type of receptor; for example, some programs appear to perform better on hydrophobic vs. hydrophilic pockets [15]. Based on these previous observations, we were interested to see how a range of academic and commercial docking programs performed when modeling CD38.

The scoring functions (SFs) and search algorithms of the programs used in this study are outlined in Table 1. Scoring functions are typically divided into three main classes: (1) physics-based SFs, which typically rely on a force-field based approach taken from molecular mechanics, (2) empirical SFs which use statistical regression techniques to fit (sometimes arbitrary) functions to experimental data (such as *K_i_/K_d_* or IC_50_ values), and (3) knowledge-based SFs, which use other data, such as covalent bond lengths alongside statistical distributions of interatomic distances determined from numerous crystal structures [14]. In practice, many scoring functions use a combination of approaches. For example, AutoDock 4 (AD4) largely uses a physics-based scoring function, with several terms taken from the Amber family of force-fields; however, these terms are scaled to fit experimentally determined binding constants, which means AD4 could be considered to use a hybrid physics-based/empirical SF [16]. In contrast, AutoDock Vina (referred to hereafter as ’Vina’) uses an empirical scoring function composed of a five-term piece-wise linear potential (PLP); however, the inclusion of atomic Van der Waals radii in the SF means Vina could be classified as a hybrid empirical/knowledge-based SF [17]. The use of PLPs in empirical scoring functions is common since fitting constant scaling values for each of the terms could be achieved simply with multiple linear regression.

Many different approaches have been taken when developing search algorithms. Although the technical aspects of these methods are outside the scope of this paper, here we give a brief overview in the context of the programs included in this benchmark study. Popular methods include genetic algorithms (used in AD4, Gold, Molegro, rDock), particle swarm optimization methods (such as the ant colony optimization method used in PLANTS, described in detail by Korb et al. [18]), simulated annealing (rDock), as well as approaches which sample promising ligand poses and perform a local optimization (as seen in Glide or Vina). In practice, search algorithms combine a variety of techniques to optimize the binding mode.

Although much work has been dedicated to improving the accuracy of scoring functions, as well as the speed and exhaustiveness of search algorithms, docking still faces fundamental challenges that hinder its applicability. The assumption that a single binding pose has enough information to reproduce the affinities, either in relative or absolute terms, rarely holds in practice. For this reason, more accurate, ensemble-based free energy scoring methods, such as molecular mechanics generalized Born and surface area continuum solvation (MM/GBSA), are routinely used to re-score compounds during virtual screening [19]. However, these methods are more computationally expensive than docking, usually making them prohibitive for large scale compound database screening.

**Figure 2:**
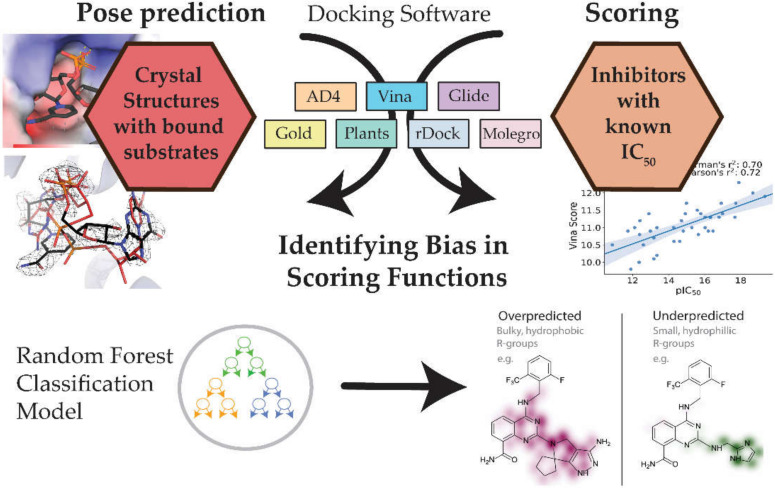
A summary of the of aims of this study, and a simplified workflow of the steps taken towards addressing these aims.

Due to the outstanding interest in the continued development of CD38 inhibitors, we were interested in benchmarking the performance of available molecular docking software as a tool for rational drug discovery for this seemingly elusive target. A simplified overview of the study is given in Figure 2. Here, seven molecular docking programs are evaluated in terms of their scoring power and ability to reproduce crystal structures: three commercial programs (Glide, Gold, and Molegro) and four academic programs (AutoDock 4, AutoDock Vina, PLANTS, and rDock). To help generalize these results for the average reader, and to allow for a fair comparison, the performance of these programs is based on their default parameters. Fortunately, the availability of structural data, in the form of crystal structures of CD38 in complex with a diverse range of biological ligands (shown in Table 2 and Figure S1), allowed us to investigate the accuracy of the docking pose predictions. We note that the biomolecule ligands in this test set are generally larger and more flexible than small molecule drug candidates, so we are cautious about suggesting that the interpretation of these results would necessarily translate to a virtual screening context. Previous efforts at determining experimental affinities and structure-activity relationship (SAR) studies have also provided suitable data to critically appraise the performance of docking programs for this target. Here, data from Deaton et al., which reports the activities for a range of strong and weak affinity binders, is used to assess the scoring and ranking power of these programs (see Table S1).

## 2. Results

### 2.1. Pose Prediction

**Figure 3:**
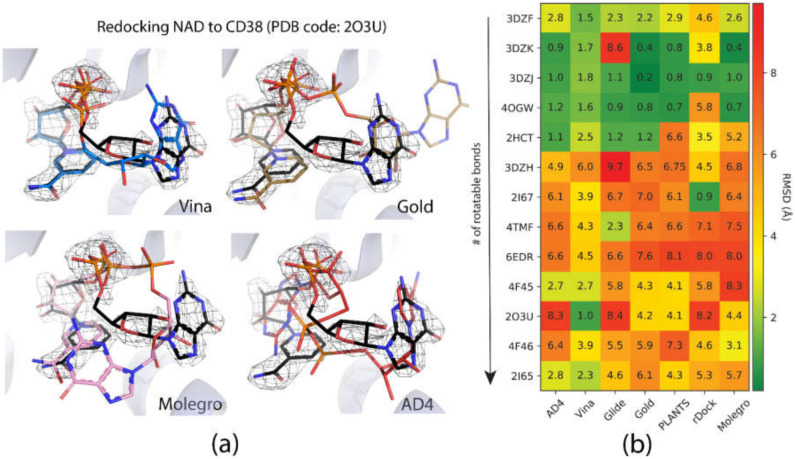
Comparisons of the redocking performance of all programs across the CD38 test set. (**a**) An example of the results produced when redocking NAD to a crystal structure of CD38 (PDB code: 2O3U). The native pose and isomesh of the electron density at 2σ in the 2f_c_–f_o_ map are shown in black. Poses were selected to be representative of the average RMSD across ten trials. In the case of all programs, the orientation of the nicotinamide fragment (essential to the catalytic mechanism of CD38) was successful, apart from AutoDock 4, which did not reproduce this feature. (**b**) A heatmap indicating the average RMSD for each program across all CD38 structures in the test set is shown. Models are sorted on the y-axis by the number of rotatable bonds.

The performance of the docking programs, when predicting the crystallographic pose of the complex, varied massively across programs and structures in the test set (see Figure 3a). Due to the diversity of the ligands in the test set, we chose to include the median root mean square deviation (RMSD) (Å) as an additional measure of re-docking success between programs. Many benchmarking efforts report mean RMSD; however, this statistic can be misleading when working with structures of varying size and flexibility [20]. The number of rotatable bonds found in the ligands in the test set ranged from three to thirteen flexible connections. We also note that using the typical 2 Å RMSD cut-off for docking ‘accuracy’ may not be appropriate for all ligands in the test set. For instance, 3 Å RMSD may be a poor result for a small molecule; however, the same result might be indicative of a good reproduction of the pose for a larger compound, such as a dinucleotide, which needs to explore a much larger conformational space. For this reason, we also present the distribution of the RMSD results for all structures in the test set (shown using the bar plots in Figure 3b).

Based on these data, Vina was identified as the program that was most consistent at finding suitable poses, giving a median RMSD of 2.5 Å over the test set, with average RMSD results ranging from 1.0 to 6.0 Å RMSD. Autodock 4 produced comparable results, giving a median RMSD for 2.8 Å over the test set. Despite this, the range in results was considerably larger (between 0.98.3 Å). Both programs were able to find a pose within 2.0 Å RMSD for approximately 35% of the test set. Vina was able to find a pose within 4.5 Å RMSD for almost 90% of structures in the test set; however, the percentage of these results in AD4 was considerably lower (approximately 55%). Gold, PLANTS, and rDock performed similarly according to these metrics, each producing a median RMSD of about 4.5 Å, with a similar range of results compared to the Autodock 4 (~ 1–8 Å RMSD).

Over the test set, Gold produced results like the native pose (within 2.0 and 4.5 Å RMSD) at a similar rate to AD4 (approximately 30% and 55%, respectively). PLANTS performed slightly worse when finding structures within 2.0 Å RMSD. Glide performed slightly worse than these programs, and, in some examples, Glide failed to find a top-scoring pose that reproduced the crystal structure. Glide produced a median docking result of 5.5 Å RMSD and could only reproduce the crystallographic pose in approximately 20% of structures. Molegro, using the MolDock GRID scoring function, performed similarly. The median RMSD of the redocked poses produced using this program was 5.2 Å.

Previous redocking studies involving CD38 have also encountered issues when reproducing crystallographic poses [7,8,9,12]. Redocking in the presence of active-site water molecules has been suggested as a method to improve the fit between the docked and the crystallographic pose. Indeed, solvent-mediated interactions have been shown to be relevant to CD38 activity [8,12]. Certainly, our poor results in the redocking section of this study are likely to be affected by challenges in modeling the ligand-solvent interactions. Unfortunately, crystallographic waters are rarely ever resolved to atomic precision. Furthermore, many scoring functions (e.g., Vina, Glide) were parameterized in the absence of solvent, and including solvation effects in docking is a known challenge [21]. Future directions aimed at assessing the performance of docking programs, which specifically model solvation effects, in the context of CD38, are recommended.

### 2.2. Scoring and Ranking Known Inhibitors

The ability of each docking program to accurately score and rank the existing dataset of forty-two inhibitors with known affinities (IC_50_) was released by Deaton et al. [22]. Pearson’s correlation coefficient, for the regression between the experimental pIC_50_ and the docking score, is reported as a measure of the “scoring accuracy”. We also present Spearman’s correlation coefficient, which is a statistical measure of how well the docking programs reproduce the experimental rankings of the compounds. Both statistics range from (−1, 1), where coefficients greater than zero represent a positive trend, and coefficients less than zero represent an inverse relationship. Some docking scores have been transformed accordingly to follow a positive trend in relation to the pIC_50_ values, so ideally, coefficients should be equal to 1.0 in the case of perfect agreement between the experiment and the modeling.

Vina and PLANTS were the top two performing programs when tested for scoring power on this data set (Pearson of 0.57 and 0.55, respectively). Despite these similar results for IC_50_ regression, Vina was markedly better at scoring the compounds than PLANTS (Spearman of 0.63 compared to 0.58). The performance of Glide and Gold was comparable, with the former being slightly better at predicting the trends in affinity (Pearson of 0.47 and 0.43, respectively), and the latter being marginally better at correctly ranking the compounds (Spearman of 0.47 and 0.43, respectively). AD4 cannot be recommended for modeling the affinities of CD38 inhibitors based on these scaffolds, as the relationship between experimental IC_50_ and docking score was no better than random chance (Pearson of 0.0). The rDock performed the worst out of all programs, producing negative Pearson’s and Spearman’s coefficients (−0.37 and −0.38, respectively), which is to say that rDock was worse than random chance at predicting the affinities for this series of compounds (see Figure S2a–g and Figure S3).

## 3. Discussion

### 3.1. Challenges with Dinucleotide Docking: Size, Flexibility, and Self-Similarity

**Figure 4:**
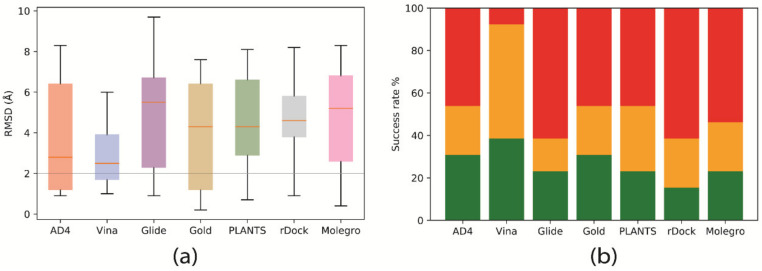
Distribution of the redocking results, and their corresponding ‘success rate’, for all programs. Only the top-scoring pose of each of the ten trials, was used to calculate the average. (**a**) A boxplot indicating the redocking accuracy in RMSD (Å). A line corresponding to the 2 Å RMSD cutoff, a typical guide for measuring redocking success, is also provided for comparison. The orange line indicates the median result for each program. (**b**) A stacked bar chart illustrating the ‘success rate’ for each of the programs. The green, orange, and red areas correspond to the percentage of models that achieved a redocking score of ≤ 2.0, ≤ 4.5, and > 4.5 Å RMSD, respectively.

The RMSD between the docked and native poses appeared to be correlated with the size and number of rotatable bonds. It has been widely acknowledged in the literature that re-docking accuracy decreases as the number of rotatable bonds increases, a phenomenon that is known to be independent of which docking program is used [23]. For the larger, dinucleotide structures in the re-docking test set, one potential challenge we predicted was that the docking programs might incorrectly guess which nucleotide fragment (i.e., nicotinamide or adenosine) sat where in the binding pocket, given the self-similarity present in the conjoined phosphoribose groups. Crystallographic evidence strongly suggests that the nicotinamide group sits inside the pocket, where π–π Van der Waals interactions with W189 keep the substrate in the correct orientation for the catalytic displacement of the nicotinamide fragment. This is also coordinated by W125, which interacts with the face of the adjacent ribose unit to further stabilize the nicotinamide fragment. For this reason, a ‘correct’ pose prediction for the dinucleotides should reproduce this important interaction. In many cases, the large values for RMSD (Figure 4b) were associated with poses that accurately predicted the position of the nicotinamide group but failed to position other parts of the molecule correctly. When re-docking the native ligand in the structure 2O3U using Gold, the average RMSD for the top-scoring pose was 4.2 Å. On inspection of the pose (see Figure 4a), the nicotinamide portion of the molecule was in good agreement with the crystallographic pose. The adenosine portion of the ligand sits outside the pocket; however, in disagreement with the experimental model, which accounts for the majority of the RMSD measured. It is possible that a more exhaustive search approach may have optimized this position of this group. Certainly, a comparison between the number of active rotatable bonds and RMSD showed that there was a statistically significant correlation between these variables for AD4, Gold, rDock, and Molgro (Figure S2a–g). This suggests that, for these programs, the default parameters for exhaustiveness may not be suitable for compounds with a high number of conformational degrees of freedom and should be increased accordingly. However, exhaustiveness many not be the only factor contributing to the poor performance of AD4 when docking dinucleotides, as the program predicted the adenosine group to sit in the same site of the nicotinamide group—the native pose—which is likely a scoring issue.

The structural disorder may also be a contributing factor in the poor performance across all docking programs for certain crystal structures, for instance, another structure of the 4′-Thioribose NAD complex (6EDR). In this case, there was considerable disorder in the adenosine ring and for W175, as indicated by the high relative B-factors of these groups in the original crystal structure. This structure models a different rotamer of W175, which faces away from the pocket. Subsequently, the adenosine group is not stabilized by W175, like in the 2O3U structure (Figure 1). For this reason, many of the programs were unable to predict the orientation of the adenosine group in a way that agreed with the original model. 3DZH appeared to be a challenge for a similar reason. The triphosphate tail was poorly resolved in the original X-ray experiment, and high B-factors for this group also suggested high uncertainty in the original model.

### 3.2. Scoring Functions Can Be Sensitive to Small Chemical Changes

**Figure 5:**
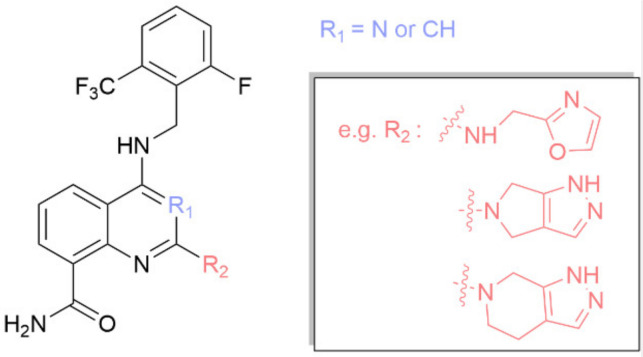
The main scaffold, and example R-groups, of the compounds screened by Deaton et al [22].

The performance of some programs (namely Vina and PLANTS) when scoring the compounds with known affinities was promising. However, we predict that chemical similarity in the dataset may contribute to bias. Most compounds in this dataset are based on the 4-amino-8-quinazoline carboxamide scaffold (R_1_ = N), shown in Figure 5, apart from two compounds containing a quinoline moiety (R_1_ = CH). Overall, the compounds in the test set were relatively similar in structure, with many R_2_ groups (examples shown in Figure 5) being nearly identical. For example, the cyclopropyl versus the cyclobutyl ring in compounds **1am** and **1an** (see Supplementary Materials Table S1). Differences in the core scaffold for the quinoline containing molecules caused some programs to drastically underpredict the affinity of these compounds. For Vina and Gold, removing the compounds containing the quinoline scaffold dramatically improved the correlation (Pearson from 0.57 to 0.72 and from 0.43 to 0.56, respectively). This suggests that a large amount of the variance observed in these data could be attributed to these poor scoring compounds. One possible explanation for this discrepancy is that the scoring function over-estimated the affinity when the atom type at this position was changed from a carbon to a hydrogen bond accepting nitrogen. Many scoring functions (Vina and Gold included) have specific terms for hydrogen bonding, which favor the addition of H-bond donors/acceptors. In contrast, removing these compounds from the Glide and PLANTS scoring test set only had a modest improvement on regression (Pearson from 0.47 to 0.49 and 0.55 to 0.60, respectively). This could be interpreted as these scoring functions being less sensitive to minor chemical changes, although more benchmarking data should be obtained to support this conclusion. Overall, while some programs were relatively successful at scoring the compounds, we expect that this result may not be generalizable to other scaffolds or drug targets. Furthermore, there is scope to investigate the role of the search algorithm parameters on the predictive powers of the docking software.

**Figure 6:**
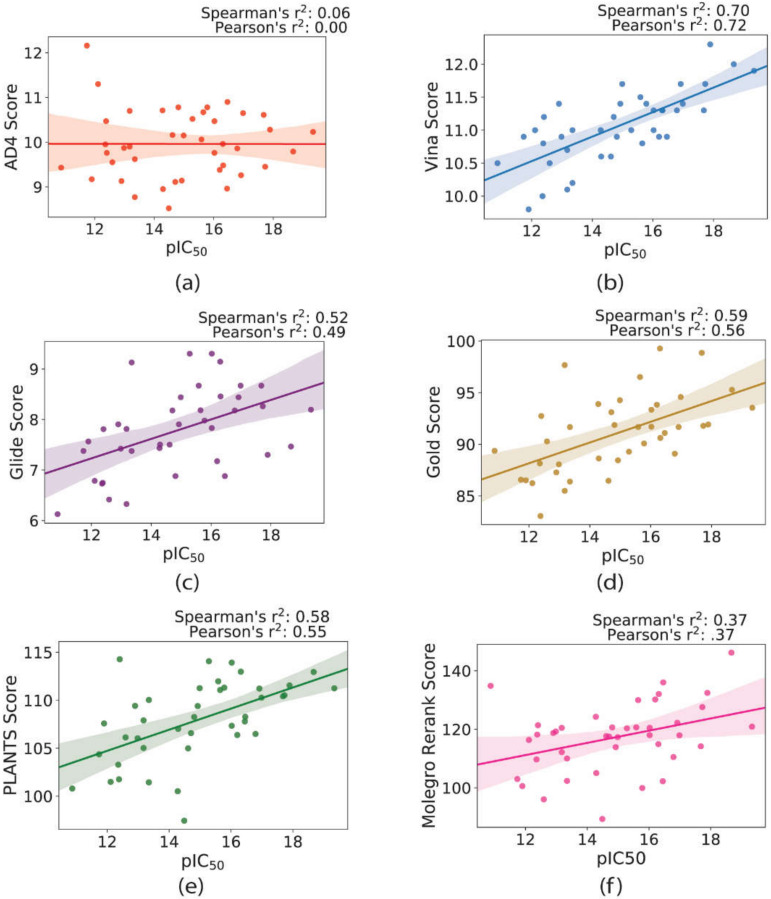
Summary of the correlation between experimental affinities (pIC_50_) for compounds screened by Deaton et al. compared to the docking score for the following programs: (**a**) AutoDock 4, (**b**) AutoDock Vina, (**c**) Glide, (**d**) Gold, (**e**) PLANTS, and (**f**) Molegro. The absolute value of the docking scores was taken to produce a positive trendline. The confidence interval for the regression estimate is shown as a translucent area centered around the line of best fit.

Among the AutoDock family, Vina outperformed AD4 in both re-docking and scoring, which agrees with previous benchmarking efforts [24,25] (see Figure 6). Overall, AD4 performed well in the re-docking trials, in comparison to other programs, however in the scoring test set, AD4 was unable to score, or rank, the compounds effectively. This observation may be unsurprising. Historically, purely physics-based SFs have been acceptable when searching for low energy binding modes, often within acceptable RMSDs from native crystal structures. However, it has been acknowledged previously that physics-based SFs are less than optimal for scoring/ranking drug candidates [26]. Due to this limitation, for example, Glide employs a final rescore of the molecular mechanics optimized poses using an empirical SF. Even though AD4’s physics-based scoring function is “calibrated” to empirical binding constants (*K_i_*/*K_d_)*, AD4’s poor scoring performance may be a symptom of its reliance on a physics-based approach.

### 3.3. Poor Pose Prediction May Lead to Poor Scoring

**Figure 7:**
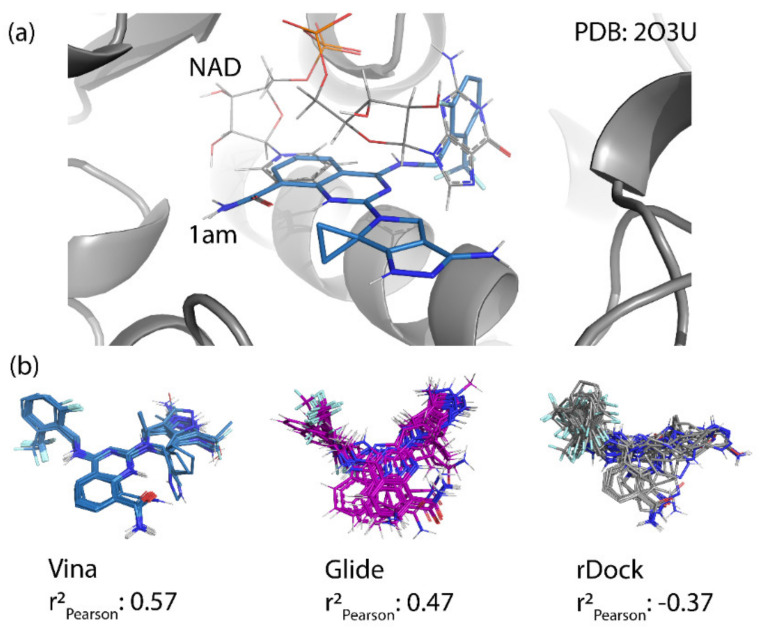
Examples of the pose predictions for some compounds from the Deaton et al. dataset. (**a**) The docked model for compound **1am** using Vina (shown in blue) compared to the native pose of NAD observed in the structure 2O3U. (**b**) Top-scoring docked poses from twenty-fix random compounds from Vina, Glide, and rDock. Despite the similar scaffolds, Glide and rDock had difficulties finding similar poses, which may have contributed to the higher variance in the predicted affinities.

Poses predicted by for Deaton’s CD38 inhibitors were generally in good agreement with the native pose of NAD. In many poses, the interactions with TRP189 were reproduced by the quinazoline moiety, which mimics the native interaction involving the nicotinamide group of NAD (see Figure 7a). Vina, Gold, Glide, and PLANTS were generally able to reproduce the position of the aromatic, adenosine group in the native pose, which is mimicked in the docked pose by the substituted aromatic ring in the CD38 inhibitors. Vina, the best performing program at scoring/ranking, produced similar poses for each of the compounds based on the alignment of the scaffold common for these compounds (see Figure 7b). This alignment was less clear in other, more poorly performing programs. By producing top-scoring poses with similar alignments, the contribution of the scaffold to the overall score should have been similar, and the scoring of each of the different R_2_ groups should have had a more dominant effect on the overall score of the compounds. We suggest that this feature of the Vina results could be one contributor to the lower variance seen over the test set. Conversely, the lack of consensus when positioning the common scaffold in other programs may produce scores with higher variance. Regardless, it is likely that this observation does not completely explain the poor scoring power of rDock, which may be the result of a pathological weakness in the rDock scoring function, at least when scoring compounds for this target.

**Figure 8:**
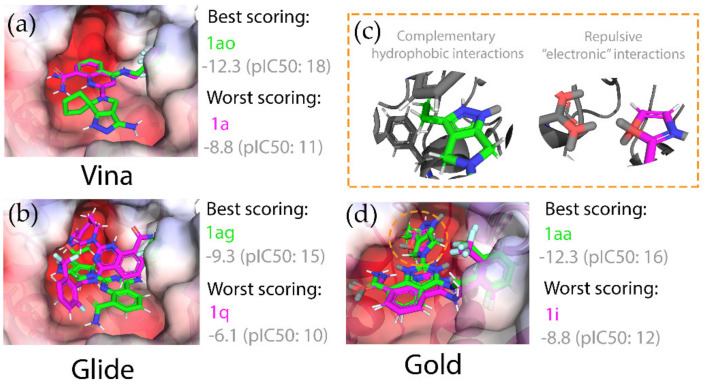
Comparisons of binding modes between the best (green) and worst (magenta) ranked compounds for (**a**) Vina, (**b**) Glide, and (**c**,**d**) Gold. In all cases, the best and worst scoring compounds were examples of low and high-affinity binders, respectively. Gold uses pseudo-atoms which represent lone pairs (for hydrogen bonding), which are represented as grey atoms. For Vina and Gold, the common scaffold of the inhibitors (Figure 5) were docked similarly. Top scoring compounds from these programs contained bulky R_2_ groups, which made favorable hydrophobic interactions with the surface of the pocket.

To draw some general observations relating pose and activity, we were interested in comparing the poses of the best and the worst scoring compounds to provide some representative examples (Figure 8). As described previously in Figure 7, the common scaffold (Figure 5) was docked similarly for most programs—parallel with the hydrophobic surface of the alpha helix. This similarity was particularly evident in Vina (Figure 8). Here, the main difference in the poses, which explains the difference in predicted score, is the position of the R_2_ group. The bulky R_2_ group of the top-scoring compound **1ao** sits comfortably in a small, hydrophobic depression in the pocket, where the cyclopentyl-moiety is well-positioned to take advantage of these Van der Waals interactions. Conversely, the small R_2_ group (R_2_ = methyl) of the lowest scoring compound **1a** is not large enough to take advantage of these interactions. The lowest scoring compound for Glide was **1q**. Here, R_2_ is too bulky (a 5,7-bicyclic ring system) to afford a pose that can sit parallel with the alpha helix, abrogating this stabilizing interaction, which seems to explain the poor score. The best and worst scoring compounds identified by Gold produced similar poses for the common scaffold. Again, favorable hydrophobic interactions of the bulk R_2_ group **1aa** were correlated with a top-scoring compound. In contrast to this, the worst scoring compound **1i** had a smaller, electrophilic R_2_ group (Figure 8). Gold uses pseudo-atoms to describe non-bonding *p* orbitals on hydrogen bonds accepting atom types (shown in grey). For **1i**, these model orbitals of the oxygen atom in the five-membered heterocyclic rings of R_2_ clash with the model orbitals of a glutamic acid residue close in space, which may be responsible for the poor score produced by Gold.

### 3.4. Random Forest to Systematically Classify Bias in Scoring Functions

**Figure 9:**
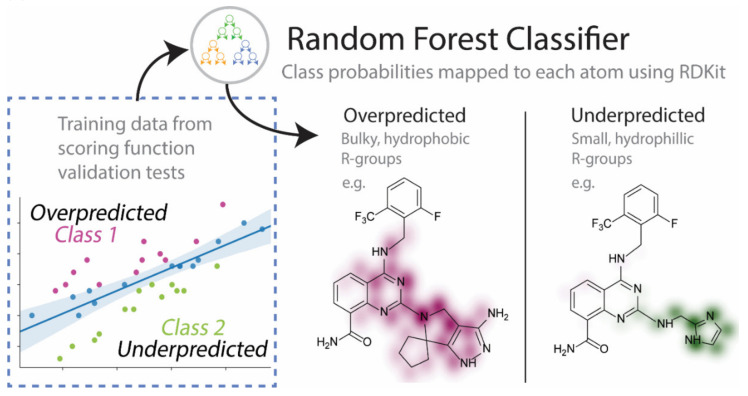
An overview of the machine learning approach used to systematically diagnose molecular features that were correlated with poor scoring accuracy. Example molecules where the scoring function performed poorly (i.e., instances where the error was larger than the 95% confidence interval) were labeled. A random forest classifier was trained on the bit vector of the Morgan fingerprint of the ligand to identify features of the ligands that may contribute to over- or underpredictions by the soring function.

Quantitative structure-activity relationship (QSAR) models in drug discovery seek to understand the relationships between high-dimensional data, such as chemical structures, and a property of interest. Machine learning models have been a popular approach to this problem. One common representation of chemical structures in these models is the molecular fingerprint, which examines the local connectivity of each atom in a structure and creates a unique identifier or “hash” to represent the atom’s local chemical environment [27]. We were interested in training a Random Forest model to identify problem chemical groups that appear to bias a scoring function to over or underpredict the affinity of a compound. A brief overview of this method is described in Figure 9. Models were trained on the fingerprints of molecules that produced errors in relative affinity outside the explained variance seen in the regression plots. The molecules in the training set were classified as “over” or “underpredicted”, and these labels were used in the training of the model. Despite their utility in machine learning methods, chemical fingerprints are difficult for humans to interpret. We used a visualization technique, available in the cheminformatics library RDKit, which maps important features of the fingerprint to the structure [28]. This is achieved by determining the change in the confidence of the classification when the portion of the fingerprint corresponding to an atom is removed. This was done for all the atoms in the structure to identify parts of the model that were strongly associated with a certain bias. 

In general, Vina tended to overpredict compounds when R_2_ was a larger, often hydrophobic substituent, and in conversely, the affinity of compounds with smaller, less hydrophobic groups at this position was underpredicted. Examples of bit-vectors taken from the molecular fingerprints used to train the model that were strongly correlated to a certain class are given in Table 3. The Vina score appeared to be biased towards lipophilic groups since the R_2_ group was generally found to dock near hydrophobic residues W189 and V185. This is illustrated by bit-vectors d and a, which correspond to a tertiary amine and a methylene carbon adjacent to a tertiary amine and an unsaturated carbon, respectively, which are common in compounds where R2 was bulky. The bit-vector corresponding to smaller R_2_ groups (Table 3, rows c and e) was identified as being correlated with underpredicted affinities. Interestingly, a simple chemical change, the addition of nitrogen to the azole ring (bit vector b), caused over predictions by Vina. This is likely because Vina has simple atom types for nitrogen, and the electronic differences (e.g., partial charge), expected when moving alchemically from a triazole to a tetrazole, are not captured by the scoring function.

Another explanation could be that smaller R_2_ groups were underpredicted, in comparison to the larger groups, due to the additive nature of scoring functions—many of which show bias towards larger substituents [29,30]. Growing a portion of a molecule to better occupy a particular site in docking will increase the docking score unless penalties are applied due to unfavorable interactions; however, the entropic penalties associated with the solvation of the larger molecule, and conformational changes to the binding pocket, are not suitably captured by SFs. When tackling the issue of bias in SFs due to the number of atoms, a “ligand efficiency” score (equal to the docking score divided by the number of atoms) can be used. The tendency for SF to over-predict affinities based on the total number of atoms was observed in all programs, except for Glide. Glide and Vina (to a lesser extent), produced a statistically significant correlation between the docking score and the ligand efficiency, which suggests that, for these programs, ligand size did not bias the scoring of these compounds (*p* values at the 95% confidence interval, using Spearman’s correlation coefficient, were 1e−11 and 0.046, respectively, see Figure S4). For virtual screening efforts, it may be beneficial to utilize two docking programs side-by-side, to account for preferences in either the scoring function. By highlighting these limitations, we hope the reader gets a better idea of the sensitivities and biases in conventional docking methods.

## 4. Materials and Methods

### 4.1. Molecular Docking

#### 4.1.1. System Selection, Search Space Definition, and Independent Trials

Available crystal structures of CD38 containing co-crystallized small molecules were obtained from the Protein Data Bank. Structures containing covalently bound ligands or multiple ligands in the binding site were removed from the test set. Certain docking programs treat ‘closed’ structures (such as rings or cyclic compounds) differently, in the interest of fairness, structures containing cyclic ligands (i.e., cyclic nicotinamide adenine dinucleotide; cNAD) were also removed from the test set. For example, many programs keep cyclic structures rigid during docking (e.g., AutoDock Vina), while some attempt to sample low energy conformations (e.g., Glide). Of the remaining structures, available electron density maps were critiqued manually to ensure the assignment of the structures of the ligand and all neighboring sidechains were supported experimentally.

A few docking programs may differ when defining the search space, usually opting to limit the position of the poses generated to lie within a rectangular prism or sphere. We opted to choose a 20 × 20 × 20 Å box (*v* = 8000 Å^3^), as this volume included all residues in the pockets and has been used in previous benchmarking efforts. When defining spherical boundary conditions, a radius of approximately 12.5 Å was used to keep the volume of the search space consistent between programs. The center of the search space was defined as the centroid of the native ligand in the crystal structure. 

Docking programs that rely heavily on stochastic processes, such as the Metropolis–Hastings algorithm in Vina or any program that uses a GA search, often produce results with large amounts of variance. All programs included in this study produced non-deterministic results, except for Glide, which appears to circumvent this behavior by performing an initial exhaustive search. Redocking experiments were performed in replicates of ten, following the protocol previous benchmarking efforts [31].

#### 4.1.2. AutoDock 4

Proteins and ligands were prepared using AutoDockTools 1.5.6. In this step, files were converted from the PDB to PDBQT format, and nonpolar hydrogens and Gasteiger charges were applied in this step. The grid points and grid point spacing were set to 60 and 0.375 Å, respectively. The parameters for the Lamarckian genetic (search) algorithm (LGA) and the scoring function were left as their default values, in correspondence with the AD4 manual.

#### 4.1.3. AutoDock Vina

Input files generated using AutoDockTools 1.5.6 were kept as is. Each trial of Vina was run using exhaustiveness of 8, while all other parameters were left as their default values.

#### 4.1.4. Glide

Proteins and ligands were prepared using the ’Protein Preparation Wizard’ in Maestro 12.0. This included the assignment of the bond order and polar and nonpolar hydrogens using the ‘Epik’ program. The Van der Waals scaling factor was set to 0.8 in accordance with the default setting, and Glide was run in standard precision (SP) mode with three poses per ligand kept.

#### 4.1.5. Gold

Preprocessed structures obtained from Maestro were used in Gold. The ‘chemscore kinase’ template was used for redocking and scoring, using Gold’s implementation of the ChemPLP scoring function, which is now defaulted. The GA search was done using 300 runs for each ligand, with all other search settings left as their default values.

#### 4.1.6. PLANTS

Proteins and ligands were prepared using SPORES version 1.3.l. PLANTS (v. 1.2) docking was performed using default parameters, with the ChemPLP scoring function, at “search speed” 1. The clustering algorithm was given a threshold of 2 Å RMSD in similarity.

#### 4.1.7. rDock

The preprocessed structures obtained from Maestro were used in rDock. rDock requires a cavity to be defined in order to run the search algorithm. The cavity was defined using the two spheres method (small sphere = 1.5 Å, large sphere = 6.0 Å) centered at the geometric center of the native ligand. Other parameters relating to the search algorithm and the scoring function were left as their default values, in correspondence with the rDock manual.

#### 4.1.8. Molegro

Protein structures were imported into the Molegro virtual docker, and solvent molecules were removed. The reference ligand of its respective protein was used as the binding site origin for redocking and scoring with a radius of 12.5 Å. The MolDock scoring function with a grid resolution of 0.30 Å was used. For other parameters, including the search algorithm and energy threshold, their default values were used.

### 4.2. Virtual Screening

Structures for the drug candidates published by Deaton et al. were sketched using ChemDraw 19.0 and imported into Maestro 12.0 as 2D, sdf formatted files. 3D coordinates for each of the ligands were generated using the ‘LigPrep’ module in Maestro 12.0. The crystal structure of CD38 (PDB code: 2O3U) was prepared used the ‘Protein Preparation Wizard’ in Maestro 12.0. This model of CD38 was chosen, based on the quality of the primary data (i.e., the electron density maps and relative B-factors), as well as its similarity (in terms of sidechain orientations in the binding site) to other published structures. This structure contains a mutation in the activate site (E226Q), which was done to aid in the crystallization of the complex and to prevent cleavage of the substrate. The ‘Mutate’ module in Maestro 12.0 was used to revert this residue back to the wildtype glutamic acid. These files were used in all other programs and prepared as necessary to meet their individual requirements.

### 4.3. Statistical Analysis and Machine Learning

Pearson and Spearman correlation calculations were performed using the functions available in the Scipy statistics library in Python 3.6. A Random Forest classifier model was build using the Scikit-Learn library in Python 3.6 and trained on the bit vector of the Morgan fingerprint (radius = 2, encoding = 2048 bits), generated using RDKit (v. 2019.09.3) for each of the drug candidates from the Deaton et al. dataset. The random forest feature weights were mapped onto the molecular graph and visualized using RDKit. Several random forest classes were generated from the data and examined individually, including high and low-affinity binders from the experimental data (cut-off IC_50_ value of 50 nm was used to determine these classes) and whether or not the docking programs over or underpredicted the affinity of the drug candidate, compared to the linear regression between docking score and IC_50_ value.

## 5. Conclusions

We examined the performance of seven docking programs for their ability to model protein-ligand interactions with CD38. The rankings for each program, when assessing pose prediction based on the median RMSD between the native and docked ligand structures, were Vina, AD4 > PLANTS, Gold, Glide, Molegro > rDock. Higher ligand flexibility appeared to negatively affect the success rate of re-docking. Docking programs were able to reproduce important π–π Van der Waals interactions with W189 in the predicted ligand poses. The rankings based on scoring power, when assessing a dataset of 42 compounds with known affinities, were: Vina, PLANTS > Glide, Gold > Molegro >> AutoDock 4 >> rDock. Out of the four performing programs for scoring, Glide had the only scoring function that was not predisposed towards favoring larger ligands. The variation in the predicted affinities, compared to the empirical data, may be influenced, in part, by variations in pose prediction between similar ligands. It is important to note that these conclusions are in the context of this protein and family of inhibitors and do not necessarily translate to other systems of interest. Finally, Random Forest classifiers have been shown to be a promising new tool in understanding and interpreting the biases in scoring functions.

## Figures and Tables

**Table 1:** Overview of the scoring functions and search algorithms of the programs tested.

Program	Scoring Function	Search Algorithm
AutoDock Vina	Empirical + Knowledge-based	Random perturbation (Metropolis–Hastings), local optimization (BFGS)
PLANTS	Empirical	Ant colony optimization
Gold	Empirical + Physics-based	Genetic algorithm (GA)
Glide	Empirical + Physics-based	Hierarchical search, local optimization
Molegro	Empirical + Physics-based	Guided differential evolution (DE)
AutoDock 4 (AD4)	Empirical + Physics-based	Lamarckian Genetic algorithm (LGA)
rDock	Empirical	GA, simulated annealing Simplex minimization

**Table 2:** A list of structures used to investigate the performance of pose prediction. These ligands, which are predominantly biologically relevant substrates for CD38, are generally larger and more flexible in comparison to the drug-like compounds commonly docked during virtual screening.

Ligand		Number of Active Rot. Bonds	PDB
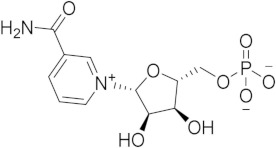	Beta-nicotinamide ribose monophosphate	4	3DZK 3DZJ 4OGW 2HCT
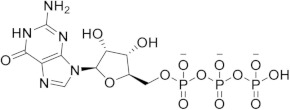	Guanosine-5′-triphosphate	8	3DZH
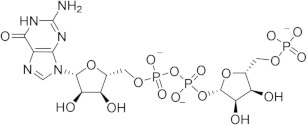	Adenosine-5-diphosphoribose	9	2I67
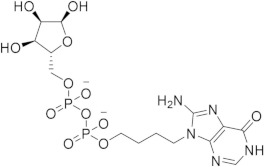	8-Amino-9- (5-*O*-butyl-diphosphoribose)-1,9-dihydro-6H-purin-6-one	11	4TMF
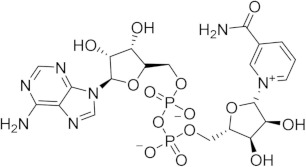	Nicotinamide adenine dinucleotide (NAD)	10	2O3U 2I65
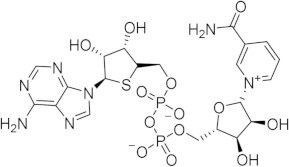	NAD (4′-Thioribose)	10	6EDR
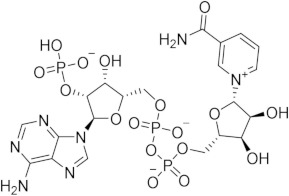	NAD (2′-*O*-phosphoric acid)	11	4F45

**Table 3:** Examples of important bit vectors of a molecular fingerprint(s) which were identified as features indicative of compounds with affinities that were over- or underpredicted by the Vina scoring function.

**Bit**		**Feature Weight**	**Class**
a	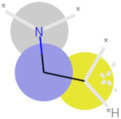	0.0206	Overpredicted
b	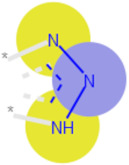	0.0205	Overpredicted
c	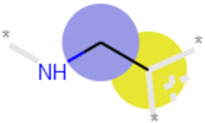	0.0201	Underpredicted
d	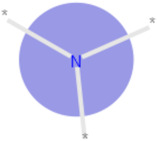	0.0190	Overpredicted
e	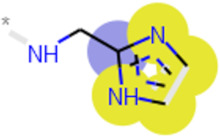	0.0089	Underpredicted

## Supplementary Materials

Supplementary materials can be found at https://www.mdpi.com/1422-0067/21/15/5183/s1. Figure S1: superposition of ligands in the active site; Figure S2: correlation between number of active rotatable bonds and RMSD: (a)Auto Dock4; (b) Vina; (c) Glide; (d) Gold; (e)PLANTS; (f) Molegro; (g) rDock; Table S1: structures of inhibitors with known affinities; Figure S3: rDock scoring performance; Figure S4: correlation between ligand efficiency and docking score.

## Abbreviations

NAD	nicotinamide adenine dinucleotide
cNAD	Cyclic nicotinamide adenine dinucleotide
CD38	cluster of differentiation 38 protein
ADPR	adenosine diphosphate ribose
cADPR	cyclic adenosine diphosphate ribose
CoMFA	Comparative Molecular Field Analysis
CASTp	Computer Atlas of Surface Topography of Proteins
AD4	AutoDock 4
SAR	Structure-activity relationship
SF	scoring function
PLP	piece-wise linear potential
GA	genetic algorithm
RMSD	root mean square deviation
MM/GBSA	Molecular mechanics generalized Born and surface area continuum solvation
QSAR	quantitative structure–activity relationship
D	Glutamic acid
K	Lysine
R	Arginine
W	Tryptophan
