# Supplementary material for: Assessing Molecular Docking Tools to Guide Targeted Drug Discovery of CD38 Inhibitors
Source: Int J Mol Sci. 2020 Jul 22;21(15):5183. doi: 10.3390/ijms21155183 (PMC7432575; doi:10.3390/ijms21155183)
Supplement: Supplementary file 1 [file ijms-21-05183-s001.pdf]

International Journal of
Molecular Sciences

MDPI

Supplementary Materials

Assessing Molecular Docking Tools to Guide Targeted Drug Discovery of CD38 Inhibitors

Eric D. Boittier [1], Yat Yin Tang [1,2], McKenna E. Buckley [2], Zachariah P. Schuurs [1,3], Derek J. Richard [1] and Neha S. Gandhi [1,3,*]

[1] Cancer & Ageing Research Program, Institute of Health and Biomedical Innovation at the Translational Research Institute (TRI), Queensland University of Technology (QUT), Brisbane, Queensland 4102, Australia; boittier@qut.edu.au (E.D.B.); yatyin.tang@connect.qut.edu.au (Y.Y.T.); zachariah.schuurs@hdr.qut.edu.au (Z.P.S.); derek.richard@qut.edu.au (D.J.R.)

[2] School of Chemistry and Physics, Faculty of Science and Engineering, Queensland University of Technology, Brisbane, QLD 4000, Australia; mckenna.buckley@connect.qut.edu.au

[3] School of Mathematical Sciences, Faculty of Science and Engineering, Queensland University of Technology, Brisbane, QLD 4000, Australia

* Correspondence: neha.gandhi@qut.edu.au; Tel.: +(61)-7-3138-7394

Received: 28 May 2020; Accepted: 14 July 2020; Published: date

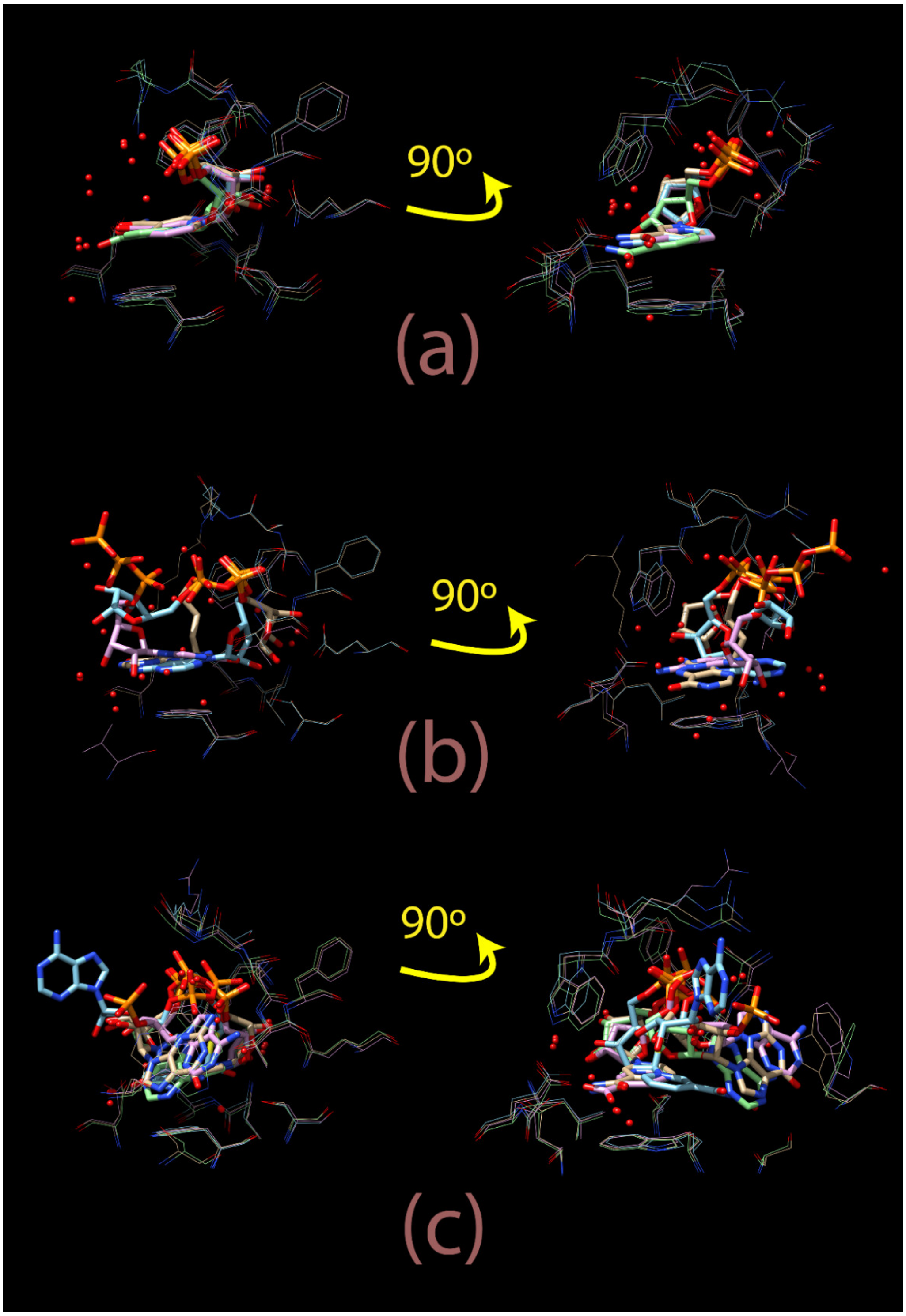

Figure S1. Superposition of a series of ligands in the active site: (**a**) beta-nicotinamide ribose monophosphate ligands (tan: 3DZK, blue: 3DZJ, pink: 4OGW, green: 2HCT), (**b**) di- and triphosphoriboses (tan: 4TMF, blue: 2I67, pink: 3DZH) and (**c**) NAD and derivatives (tan: 2O3U, blue: 6EDR, pink: 4F45, green: 2I65). Water is shown as red spheres.

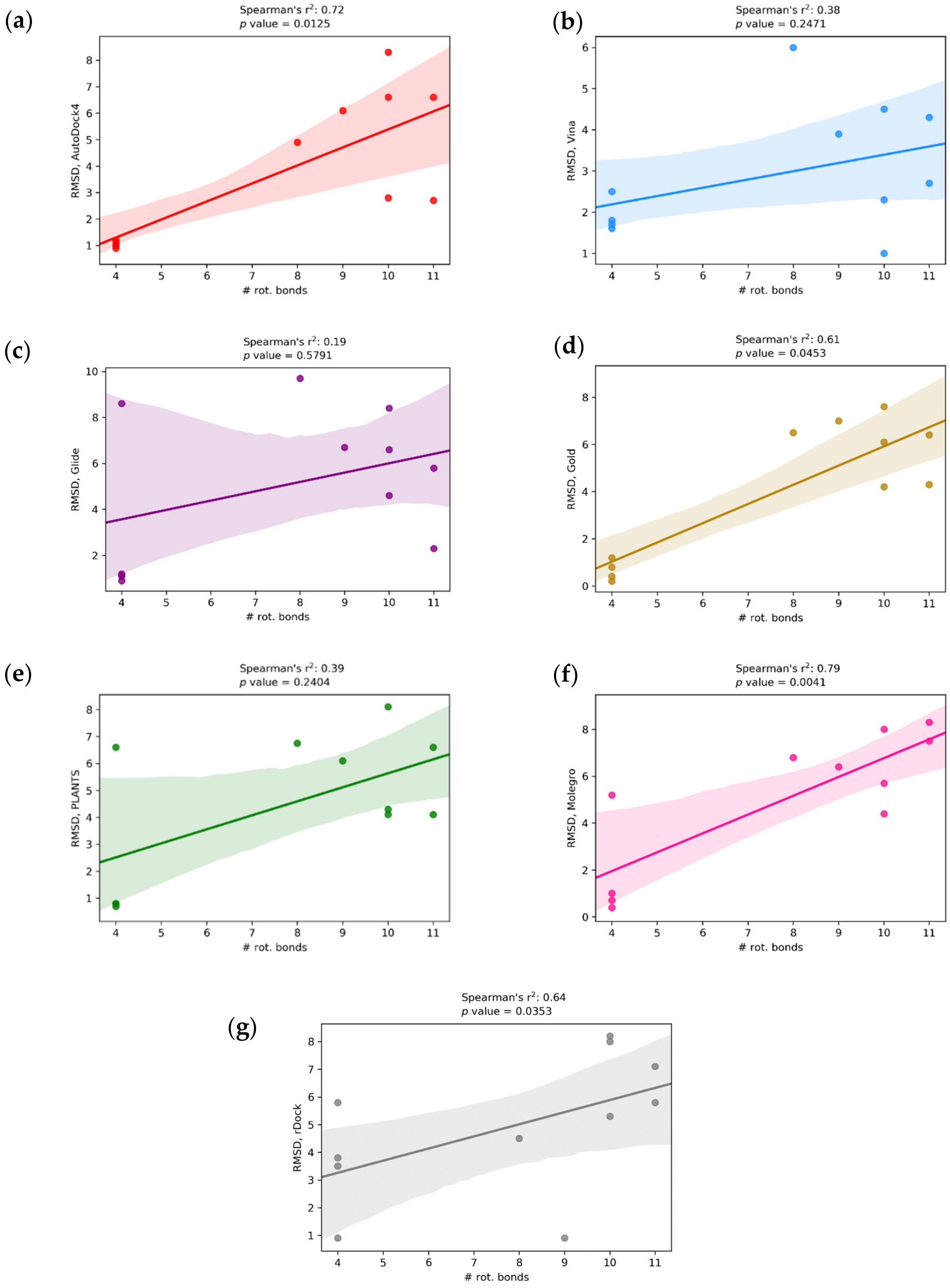

Figure S2. Correlation between number of active rotatable bonds and RMSD for the programs like (**a**) AutoDock 4, (**b**) Vina, (**c**) Glide, (**d**) Gold, (**e**) PLANTS, (**f**) Molegro and (**g**) rDock assessed in this study. Spearman's rank correlation and associated *p* values at the 95% confidence interval are reported (here, the null hypothesis is that there is a correlation). Based on these limited results, AutoDock4, Gold, Molegro and rDock appeared to be particularly affected with ligands with high conformational degrees of freedom.

Table S1. Structures of ligands used to assess scoring power.

Chemical Formula	Structure	IC50 nM	S.D	Number
$C_{19}H_{15}F_4N_3O$	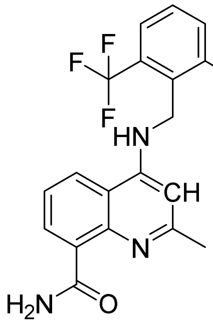	510	100	**1a**
$C_{22}H_{18}F_4N_6O$	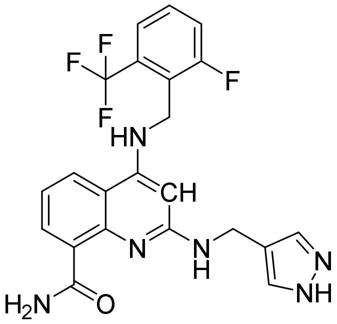	72	13	**1b**
$C_{21}H_{17}F_4N_7O$	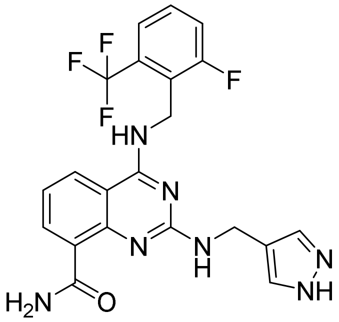	450	81	**1c**
$C_{21}H_{17}F_4N_7O$	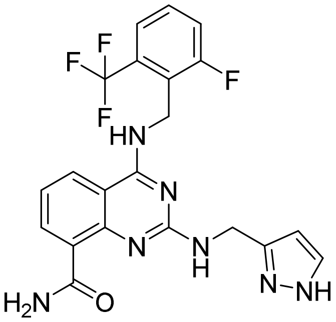	2300	37	**1d**

$C_{20}H_{16}F_4N_8O$	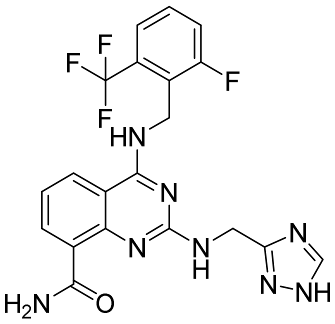	4300	210	**1e**
$C_{21}H_{17}F_4N_7O$	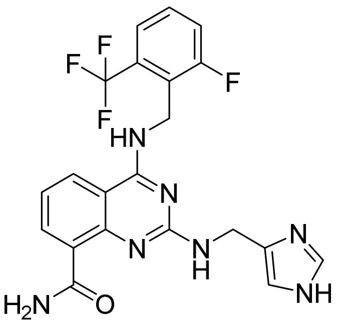	1600	610	**1f**
$C_{21}H_{17}F_4N_7O$	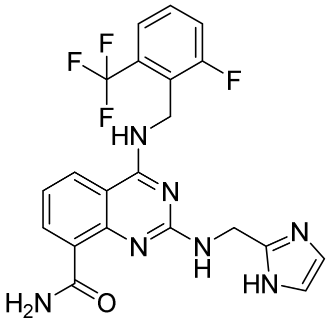	6800	4800	**1g**
$C_{19}H_{15}F_4N_9O$	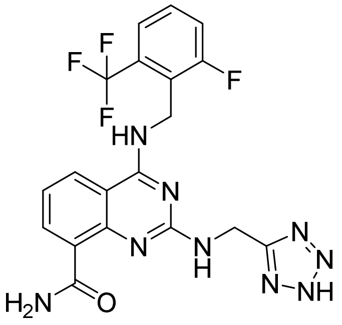	8000	2400	**1h**

$C_{21}H_{16}F_4N_6O_2$.	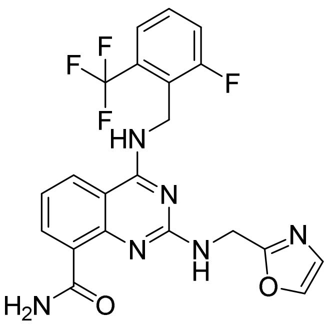	4200	200	**1i**
$C_{21}H_{16}F_4N_6O_2$	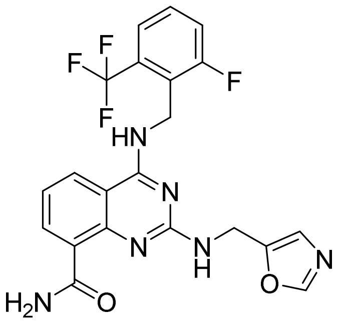	3400	1800	**1j**
$C_{21}H_{16}F_4N_6O_2$	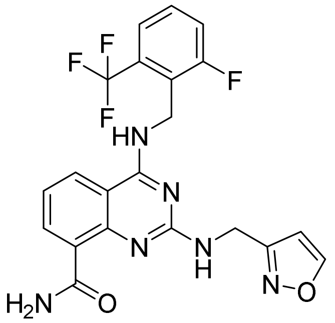	5500	1600	**1k**
$C_{22}H_{19}F_4N_7O$	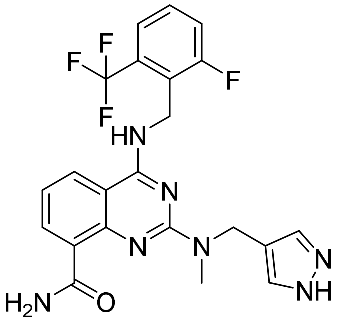	1900	120	**1l**

$C_{22}H_{19}F_4N_7O$	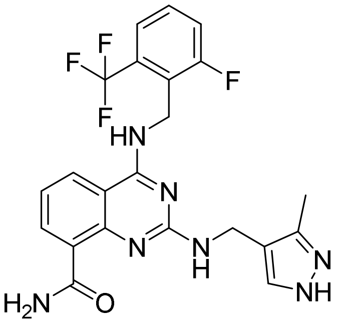	620	10	**1m**
$C_{22}H_{17}F_4N_7O$	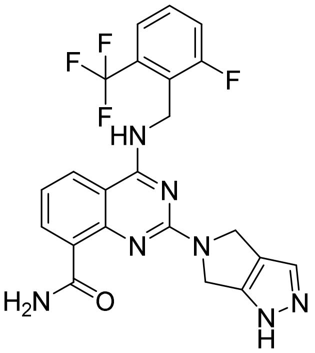	92	7.5	**1n**
$C_{23}H_{19}F_4N_7O$	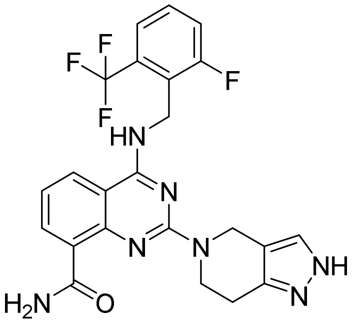	630	21	**1o**
$C_{23}H_{19}F_4N_7O$	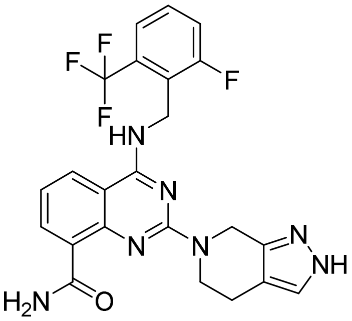	1900	390	**1p**

$C_{24}H_{21}F_4N_7O$	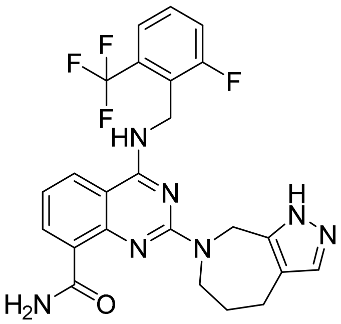	19000	1800	**1q**
$C_{23}H_{19}F_4N_7O$	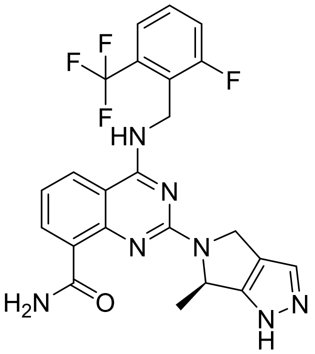	51	3.3	**1r**
$C_{23}H_{19}F_4N_7O$	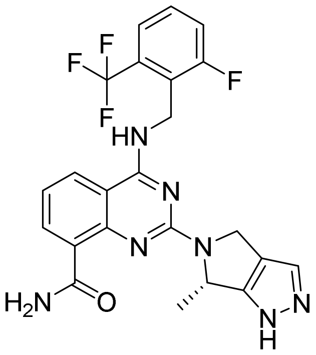	410	100	**1s**
$C_{23}H_{19}F_4N_7O$	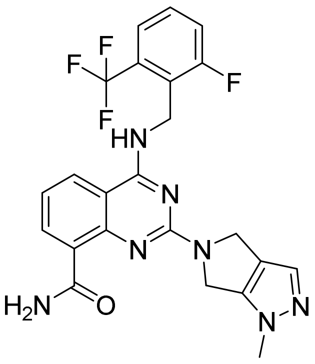	82	22	**1t**

$C_{23}H_{19}F_4N_7O$	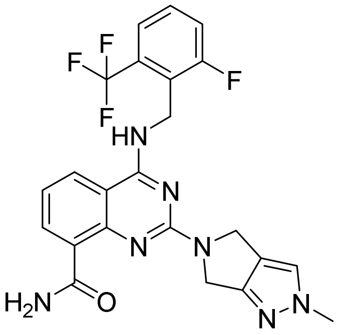	160	19	**1u**
$C_{23}H_{19}F_4N_7O$	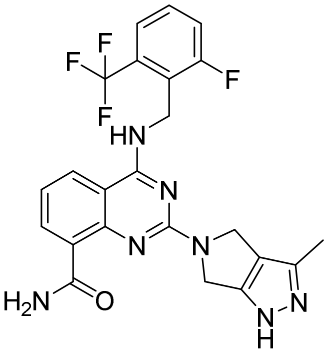	42	22	**1v**
$C_{23}H_{19}F_4N_7O$	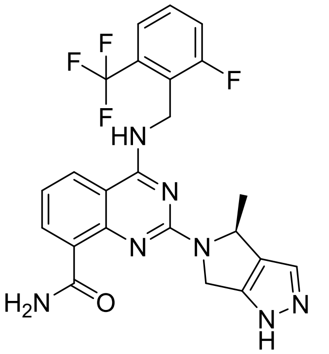	2500	120	**1w**
$C_{23}H_{19}F_4N_7O$	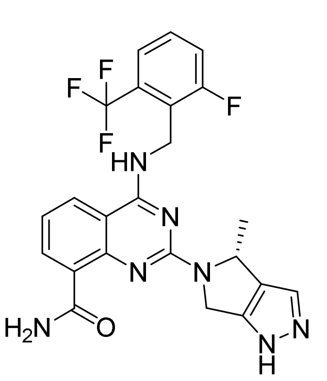	330	43	**1x**

$C_{24}H_{21}F_4N_7O$	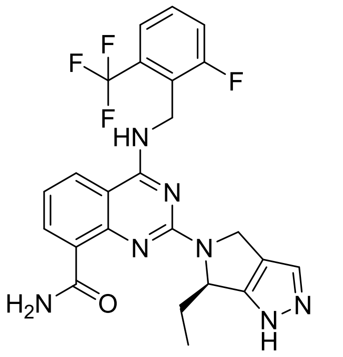	71	0	**1y**
$C_{24}H_{21}F_4N_7O$	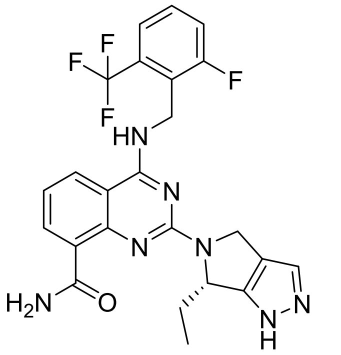	370	18	**1z**
$C_{24}H_{21}F_4N_7O$	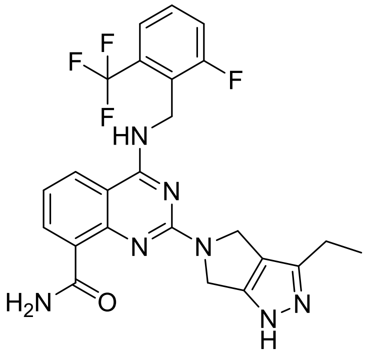	83	5.4	**1aa**
$C_{23}H_{16}F_7N_7O$	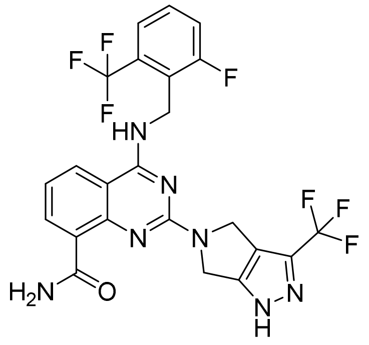	4100	200	**1ab**

$C_{22}H_{17}F_4N_7O_2$	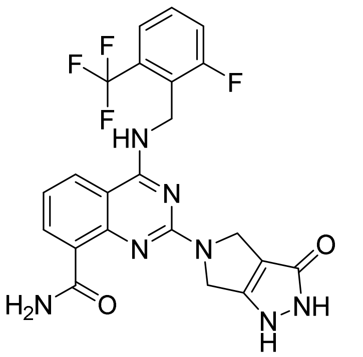	1600	320	**1ac**
$C_{22}H_{18}F_4N_8O$	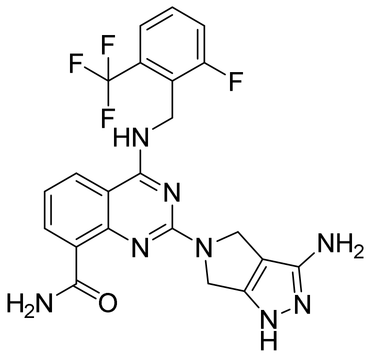	110	29	**1ad**
$C_{23}H_{18}F_4N_8O_2$	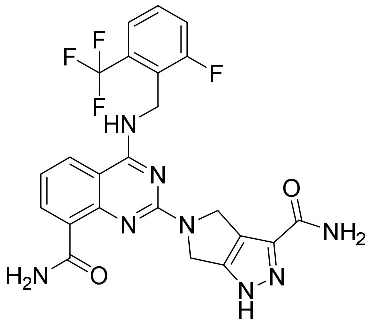	140	13	**1ae**
$C_{24}H_{21}F_4N_7O_2$	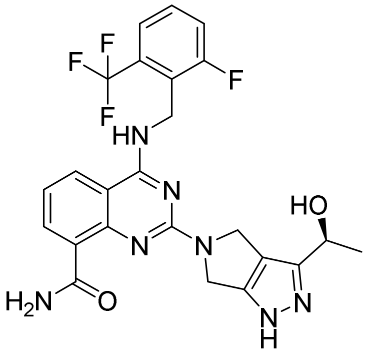	110	12	**1af**
same as 1af		230	34	**1ag**

$C_{23}H_{19}F_4N_7O$	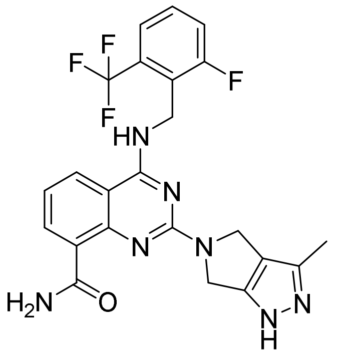	21		**1ah**
$C_{24}H_{21}F_4N_7O$	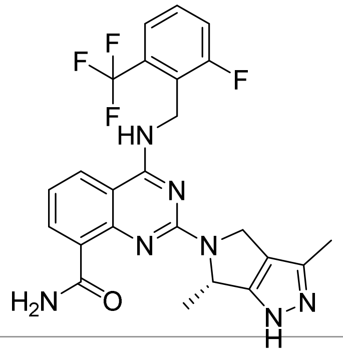	170	28	**1ai**
$C_{23}H_{20}F_4N_8O$	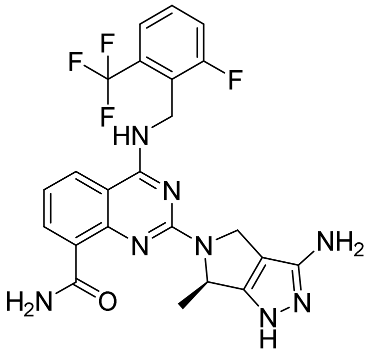	45	0	**1aj**
$C_{23}H_{20}F_4N_8O$	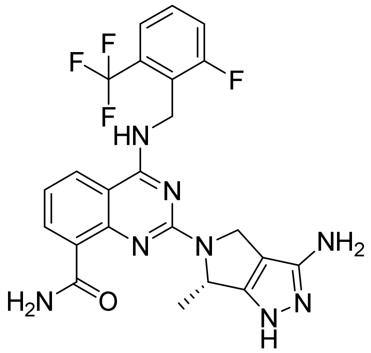	310	5	**1ak**

$C_{24}H_{22}F_4N_8O$	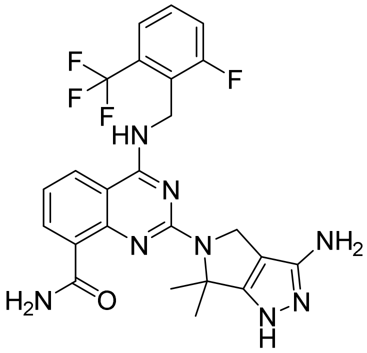	20	5.5	**1al**
$C_{24}H_{20}F_4N_8O$	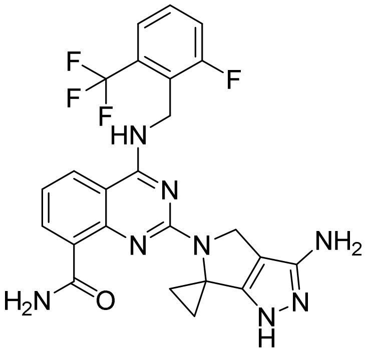	4	3.6	**1am**
$C_{25}H_{22}F_4N_8O$	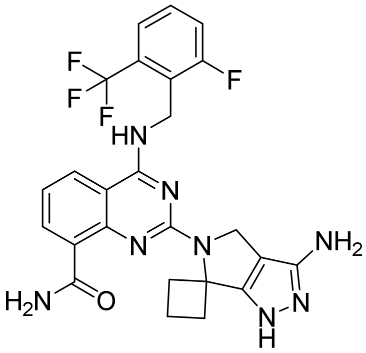	7.8	0.76	**1an**
$C_{26}H_{24}F_4N_8O$	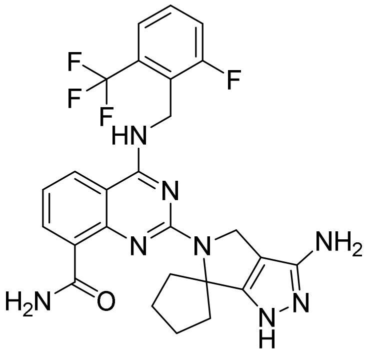	17	0.54	**1ao**

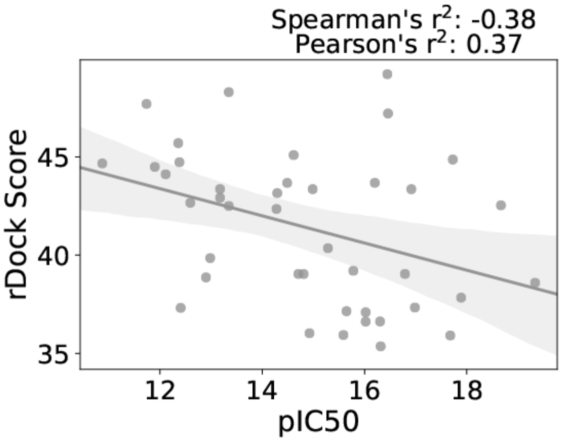

Figure S3. rDock scoring performance.

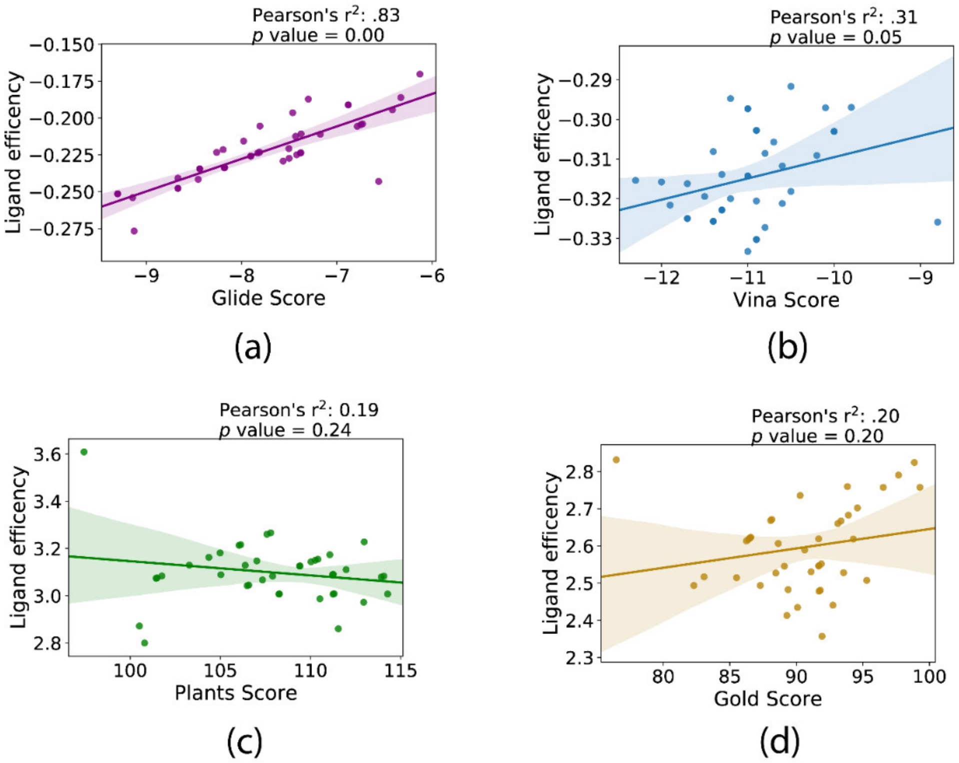

Figure S4. Analysis of additive, "ligand" bias (bias towards better scoring as number of heavy atoms increases) observed in the top performing programs in the scoring power tests. Ligand efficiency (in this context, the score divided by number of heavy atoms) can be used as a measure of "ligand" bias. For a docking score that correlates with ligand efficiency, it is assumed that there is no ligand bias. Pearson's correlation and associated p values at the 95% confidence interval are reported (here, the null hypothesis is that there is a correlation between ligand efficiency and docking score) for (**a**) Glide, (**b**) Vina, (**c**) Plants and (**d**) Gold scores. Plants and Gold showed no correlation between ligand efficiency and docking score, which suggests that the scoring functions may be subject to ligand bias. Glide showed a strong correlation. Vina showed a small, but statistically significant, correlation between ligand efficiency.
